# 2-(4-Methyl­phen­yl)-6-nitro-1,3-benzoxazole

**DOI:** 10.1107/S1600536813008970

**Published:** 2013-04-05

**Authors:** Roberto Centore, Vincenzo Piccialli, Angela Tuzi

**Affiliations:** a Dipartimento di Scienze Chimiche, Università degli Studi di Napoli ’Federico II’, Complesso di Monte S. Angelo, Via Cinthia, 80126 Napoli, Italy

## Abstract

The title compound, C_14_H_10_N_2_O_3_, is a π-conjugated mol­ecule containing a benzoxazole aromatic fused heterobicycle. The benzoxazole ring system is planar within 0.01 Å. The mol­ecule assumes an approximately flat conformation, the benzoxazole ring system forming dihedral angles of 6.52 (12) and 7.4 (3)° with the benzene ring and the nitro group, respectively. In the crystal, mol­ecules are connected by very weak C—H⋯O hydrogen inter­actions, forming chains running parallel to the *a* or *c* axes. The methyl H atoms are disordered over two sets of sites of equal occupancy rotated by 60°.

## Related literature

For general information on heterocycles in organic electronics and optoelectronics, see: Dalton (2002 ▶); Heeger (2010 ▶). For heterocycle-based semiconductors, optoelectronic and piezoelectric materials, see: Carella, Centore, Sirigu *et al.* (2004 ▶); Centore, Ricciotti *et al.* (2012 ▶); Centore, Concilio *et al.* (2012 ▶). For structural and theoretical analysis of conjugation in heterocycle-based organic mol­ecules, see: Carella, Centore, Fort *et al.* (2004 ▶); Gainsford *et al.* (2008 ▶). For structural and theoretical analysis of conjugation in heterocycle-based metallorganic compounds, see: Takjoo *et al.* (2011 ▶); Takjoo & Centore (2013 ▶). For theoretical computations on similar compounds, see: Capobianco *et al.* (2012 ▶, 2013 ▶). For the synthesis of related heterocyclic compounds, see: Bruno *et al.* (2002 ▶); Centore *et al.* (2007 ▶); Piccialli *et al.* (2013 ▶); Centore, Fusco, Capobianco *et al.* (2013 ▶). For hydrogen bonding in crystals see: Desiraju & Steiner (1999 ▶); Centore, Fusco, Jazbinsek *et al.* (2013 ▶).
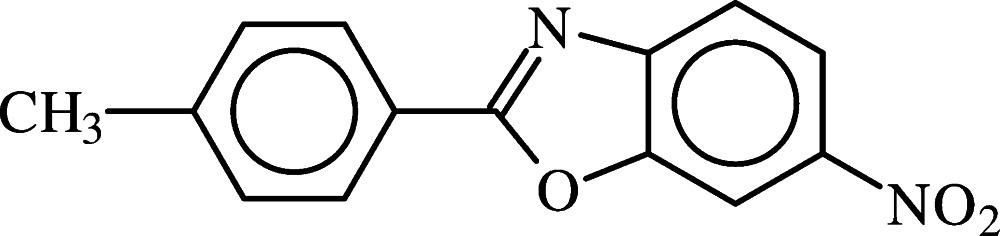


## Experimental

### 

#### Crystal data

C_14_H_10_N_2_O_3_
*M* *_r_* = 254.24
Orthorhombic, 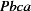

*a* = 27.251 (4) Å
*b* = 7.4457 (6) Å
*c* = 11.990 (9) Å
*V* = 2432.8 (19) Å^3^
*Z* = 8
Mo *K*α radiation
μ = 0.10 mm^−1^
*T* = 293 K
0.40 × 0.20 × 0.20 mm

#### Data collection

Enraf–Nonius MACH3 diffractometer
2968 measured reflections
2140 independent reflections
970 reflections with *I* > 2σ(*I*)
*R*_int_ = 0.020
1 standard reflections every 120 min intensity decay: none

#### Refinement

*R*[*F* ^2^ > 2σ(*F* ^2^)] = 0.055
*wR*(*F* ^2^) = 0.168
*S* = 1.11
2140 reflections
174 parameters
H-atom parameters constrained
Δρ_max_ = 0.14 e Å^−3^
Δρ_min_ = −0.19 e Å^−3^

### 

Data collection: *MACH3*/*PC* Software (Nonius, 1996 ▶); cell refinement: *CELLFITW* (Centore, 2004 ▶); data reduction: *XCAD4* (Harms & Wocadlo, 1995 ▶); program(s) used to solve structure: *SIR97* (Altomare *et al.*, 1999 ▶); program(s) used to refine structure: *SHELXL97* (Sheldrick, 2008 ▶); molecular graphics: *ORTEP-3 for Windows* (Farrugia, 2012 ▶) and *Mercury* (Macrae *et al.*, 2006 ▶); software used to prepare material for publication: *WinGX* (Farrugia, 2012 ▶).

## Supplementary Material

Crystal structure: contains datablock(s) global, I. DOI: 10.1107/S1600536813008970/rz5053sup1.cif

Structure factors: contains datablock(s) I. DOI: 10.1107/S1600536813008970/rz5053Isup2.hkl

Supplementary material file. DOI: 10.1107/S1600536813008970/rz5053Isup3.cml

Additional supplementary materials:  crystallographic information; 3D view; checkCIF report

## Figures and Tables

**Table 1:** Hydrogen-bond geometry (Å, °)

*D*—H⋯*A*	*D*—H	H⋯*A*	*D*⋯*A*	*D*—H⋯*A*
C10—H10⋯O2^i^	0.93	2.61	3.502 (6)	160
C1—H1*B*⋯O2^ii^	0.96	2.80	3.143 (5)	102

Symmetry codes: (i) 
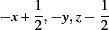
; (ii) 
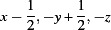
.

**Fig. 1.**
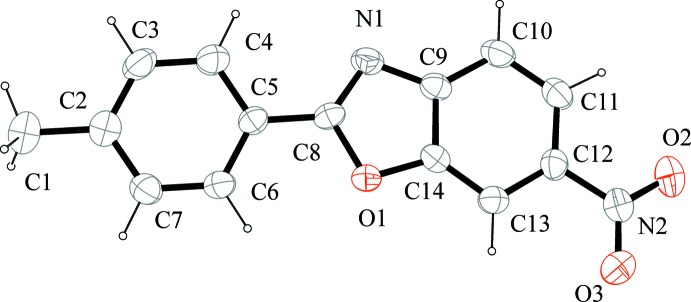
ORTEP view of the molecular structure of the title compound. Thermal ellipsoids are drawn at 50% probability level. Only one orientation of the disordered methyl group is shown.

**Fig. 2.**
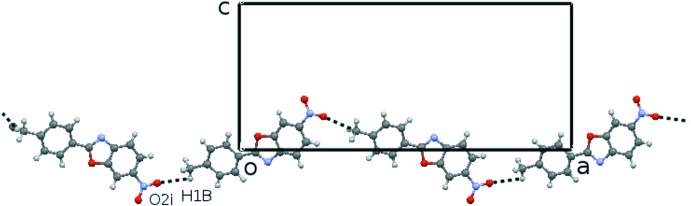
Row of molecules of the title compound running along the a axis. Only one orientation of the disordered methyl group is shown. Hydrogen bonds are shown as dashed lines.

**Fig. 3.**
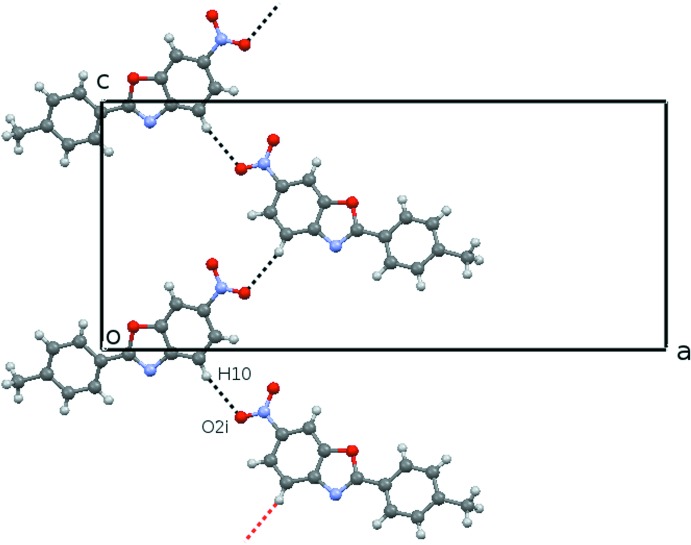
Row of molecules of the title compound running along the c. Only one orientation of the disordered methyl group is shown. Hydrogen bonds are shown as dashed lines.

## supplementary crystallographic information

### Comment

Heterocycles are important compounds of synthetic chemistry. Besides their long standing and relevant application as drugs and bioactive compounds, aromatic heterocycles are playing a fundamental role in modern material chemistry as building blocks of conjugated active molecules in some emerging fields of organic electronics and optoelectronics: conducting polymers and organic solar cells (Heeger, 2010), organic field-effect transistors (Centore, Ricciotti *et al.*, 2012), nonlinear optically active and piezoelectric compounds (Dalton, 2002; Carella, Centore, Sirigu *et al.* (2004); Centore, Concilio *et al.*, 2012). The chemical investigation is mainly directed to the synthesis of new molecules or conjugated polymers containing heterocyclic moieties. However, also the structural investigation of the molecules is relevant, pointing towards the quantitative evaluation of the structural parameters related to the conjugation (Carella, Centore, Fort *et al.*, 2004; Gainsford *et al.*, 2008; Capobianco *et al.*, 2012; Capobianco *et al.*, 2013). Following our interest in the synthesis and characterization of new heterocyclic compounds, including metal containing heterocyclic compounds (Takjoo *et al.*, 2011; Takjoo & Centore, 2013) for applications as advanced materials and bioactive compounds, and in the analysis of crystal structures controlled by the formation of H bonds (Centore, Fusco, Jazbinsek *et al.*, 2013), we report, in the present paper, the structural investigation of the title compound, shown in Scheme 1. 2-(4-methyl)-phenyl-6-nitro-benzoxazole is an organic dye containing the 6-nitrobenzoxazole acceptor group conjugated with a 4-methylphenyl moiety. The 6-nitrobenzoxazole-2-yl moiety has been used in the synthesis of polymers showing quadratic NLO behaviour (Bruno *et al.*, 2002).

The molecular structure of the title compound is shown in Fig. 1. The phenyl and benzoxazole rings are nearly coplanar, the dihedral angle between the mean planes being 6.7 (1)°. That structural feature is in accordance with the expected π conjugation of the compound.

Molecules in the crystal form rows through very weak hydrogen interactions between methyl or aromatic C–H donors and oxygen acceptors of the nitro group (Fig. 2 and Fig. 3; Table 1). The chains, which have graph set symbol *C*^1^_1_(14) and *C*^1^_1_(7) are generated, respectively, by the *b* and *a* glide planes.

### Experimental

The title compound was prepared by reaction of 2-amino-5-nitrophenol (5.00 g, 32.4 mmol) with toluic acid (4.41 g, 32.4 mmol) in polyphosphoric acid (150 g) at 150°C. The dehydration procedure is analogous to that we have already described for the synthesis of similar chromophores (Bruno *et al.*, 2002; Centore *et al.*, 2007). Purification of the title compound was obtained by recrystallization from ethanol. The final yield was 5.69 g (69%). M. p. 437 K. Single crystals were obtained by slow evaporation of an ethanol solution. ^1^H-NMR (CDCl_3_) δ 2.47 (s, 3H), 7.38 (d, 2H, J = 7.9 Hz), 7.82 (d, 1H, J= 8.5 Hz), 8.18 (d, 2H, J = 8.3 Hz), 8.33 (d, 1H, J= 8.7 Hz), 8.48 (d, 1H, J= 1.8 Hz).

### Refinement

All H atoms were generated stereochemically. In particular, the methyl group is disordered over two sets of sites of equal occupancy rotated from each other by 60°. All H atoms were refined by a riding model with C—H = 0.93-0.96 Å and with *U*_iso_(H) = 1.2*U*_eq_(C) or 1.5*U*_eq_(C) for methyl hydrogen atoms.

### Crystal data

**Table:**

C_14_H_10_N_2_O_3_	*F*(000) = 1056
*M**_r_* = 254.24	*D*_x_ = 1.388 Mg m^−^^3^
Orthorhombic, *P**b**c**a*	Mo *K*α radiation, λ = 0.71073 Å
Hall symbol: -P 2ac 2ab	Cell parameters from 25 reflections
*a* = 27.251 (4) Å	θ = 12.2–12.4°
*b* = 7.4457 (6) Å	µ = 0.10 mm^−^^1^
*c* = 11.990 (9) Å	*T* = 293 K
*V* = 2432.8 (19) Å^3^	Prism, brown
*Z* = 8	0.40 × 0.20 × 0.20 mm

### Data collection

**Table:**

Enraf–Nonius MACH3 diffractometer	*R*_int_ = 0.020
Radiation source: fine-focus sealed tube	θ_max_ = 25.0°, θ_min_ = 1.5°
Graphite monochromator	*h* = −12→32
non–profiled ω scans	*k* = −3→8
2968 measured reflections	*l* = −5→14
2140 independent reflections	1 standard reflections every 120 min
970 reflections with *I* > 2σ(*I*)	intensity decay: none

### Refinement

**Table:**

Refinement on *F*^2^	Primary atom site location: structure-invariant direct methods
Least-squares matrix: full	Secondary atom site location: difference Fourier map
*R*[*F*^2^ > 2σ(*F*^2^)] = 0.055	Hydrogen site location: inferred from neighbouring sites
*wR*(*F*^2^) = 0.168	H-atom parameters constrained
*S* = 1.11	*w* = 1/[σ^2^(*F*_o_^2^) + (0.0553*P*)^2^ + 0.383*P*] where *P* = (*F*_o_^2^ + 2*F*_c_^2^)/3
2140 reflections	(Δ/σ)_max_ < 0.001
174 parameters	Δρ_max_ = 0.14 e Å^−^^3^
0 restraints	Δρ_min_ = −0.19 e Å^−^^3^

### Special details

**Table:**

Geometry. All e.s.d.'s (except the e.s.d. in the dihedral angle between two l.s. planes) are estimated using the full covariance matrix. The cell e.s.d.'s are taken into account individually in the estimation of e.s.d.'s in distances, angles and torsion angles; correlations between e.s.d.'s in cell parameters are only used when they are defined by crystal symmetry. An approximate (isotropic) treatment of cell e.s.d.'s is used for estimating e.s.d.'s involving l.s. planes.
Refinement. Refinement of *F*^2^ against ALL reflections. The weighted *R*-factor *wR* and goodness of fit *S* are based on *F*^2^, conventional *R*-factors *R* are based on *F*, with *F* set to zero for negative *F*^2^. The threshold expression of *F*^2^ > σ(*F*^2^) is used only for calculating *R*-factors(gt) *etc*. and is not relevant to the choice of reflections for refinement. *R*-factors based on *F*^2^ are statistically about twice as large as those based on *F*, and *R*- factors based on ALL data will be even larger.

### Fractional atomic coordinates and isotropic or equivalent isotropic displacement parameters (Å^2^)

**Table:**

	*x*	*y*	*z*	*U*_iso_*/*U*_eq_	Occ. (<1)
O1	0.05625 (9)	0.1644 (3)	0.0918 (2)	0.0545 (7)	
O2	0.25311 (13)	−0.0885 (6)	0.2271 (3)	0.1125 (14)	
O3	0.19587 (12)	−0.0394 (6)	0.3439 (3)	0.1144 (14)	
N1	0.08562 (12)	0.1994 (4)	−0.0827 (3)	0.0614 (10)	
N2	0.21215 (15)	−0.0363 (6)	0.2493 (4)	0.0771 (11)	
C1	−0.14836 (14)	0.4375 (6)	−0.1283 (4)	0.0819 (15)	
H1A	−0.1684	0.3329	−0.1391	0.123*	0.57 (5)
H1B	−0.1477	0.5068	−0.1958	0.123*	0.57 (5)
H1C	−0.1618	0.5090	−0.0690	0.123*	0.57 (5)
H1D	−0.1502	0.5663	−0.1302	0.123*	0.43 (5)
H1E	−0.1709	0.3923	−0.0735	0.123*	0.43 (5)
H1F	−0.1568	0.3901	−0.2002	0.123*	0.43 (5)
C2	−0.09695 (16)	0.3808 (5)	−0.0983 (4)	0.0624 (12)	
C3	−0.05929 (17)	0.3930 (6)	−0.1742 (4)	0.0754 (13)	
H3	−0.0656	0.4411	−0.2443	0.091*	
C4	−0.01233 (16)	0.3358 (6)	−0.1496 (4)	0.0710 (13)	
H4	0.0122	0.3420	−0.2035	0.085*	
C5	−0.00180 (14)	0.2695 (5)	−0.0448 (3)	0.0516 (10)	
C6	−0.03907 (14)	0.2611 (5)	0.0325 (3)	0.0621 (11)	
H6	−0.0325	0.2182	0.1038	0.074*	
C7	−0.08606 (15)	0.3154 (6)	0.0058 (4)	0.0682 (13)	
H7	−0.1107	0.3075	0.0592	0.082*	
C8	0.04769 (15)	0.2121 (5)	−0.0175 (3)	0.0521 (10)	
C9	0.12336 (14)	0.1393 (5)	−0.0134 (3)	0.0548 (11)	
C10	0.17235 (15)	0.1012 (6)	−0.0351 (4)	0.0724 (13)	
H10	0.1855	0.1146	−0.1062	0.087*	
C11	0.20086 (14)	0.0426 (6)	0.0532 (4)	0.0718 (13)	
H11	0.2337	0.0139	0.0416	0.086*	
C12	0.18069 (14)	0.0268 (6)	0.1583 (4)	0.0592 (11)	
C13	0.13235 (14)	0.0639 (5)	0.1837 (3)	0.0571 (11)	
H13	0.1194	0.0525	0.2551	0.069*	
C14	0.10530 (14)	0.1191 (5)	0.0941 (3)	0.0509 (10)	

### Atomic displacement parameters (Å^2^)

**Table:**

	*U*^11^	*U*^22^	*U*^33^	*U*^12^	*U*^13^	*U*^23^
O1	0.0553 (17)	0.0632 (18)	0.0449 (16)	0.0015 (13)	0.0038 (13)	0.0002 (14)
O2	0.057 (2)	0.171 (4)	0.110 (3)	0.020 (2)	−0.005 (2)	0.013 (3)
O3	0.086 (2)	0.190 (4)	0.068 (2)	0.022 (3)	−0.004 (2)	0.022 (3)
N1	0.064 (2)	0.073 (3)	0.0474 (19)	−0.0022 (18)	0.010 (2)	−0.0039 (19)
N2	0.058 (2)	0.096 (3)	0.077 (3)	−0.006 (2)	−0.004 (2)	0.006 (3)
C1	0.073 (3)	0.070 (3)	0.103 (4)	0.010 (3)	−0.024 (3)	−0.008 (3)
C2	0.071 (3)	0.051 (3)	0.066 (3)	0.000 (2)	−0.008 (3)	−0.011 (3)
C3	0.090 (3)	0.087 (3)	0.049 (3)	0.014 (3)	−0.011 (3)	0.007 (3)
C4	0.075 (3)	0.086 (3)	0.052 (3)	0.009 (3)	0.002 (2)	−0.001 (3)
C5	0.060 (2)	0.051 (3)	0.044 (2)	0.000 (2)	−0.002 (2)	−0.003 (2)
C6	0.064 (3)	0.073 (3)	0.050 (2)	−0.004 (3)	0.002 (2)	0.004 (2)
C7	0.060 (3)	0.078 (3)	0.067 (3)	−0.006 (2)	0.004 (2)	0.001 (3)
C8	0.067 (3)	0.051 (3)	0.038 (2)	0.000 (2)	−0.001 (2)	−0.003 (2)
C9	0.058 (3)	0.058 (3)	0.048 (2)	−0.009 (2)	0.008 (2)	−0.007 (2)
C10	0.065 (3)	0.093 (4)	0.058 (3)	−0.003 (3)	0.019 (3)	−0.001 (3)
C11	0.053 (3)	0.092 (3)	0.070 (3)	−0.001 (3)	0.010 (2)	−0.003 (3)
C12	0.050 (2)	0.066 (3)	0.062 (3)	−0.003 (2)	−0.005 (2)	−0.002 (2)
C13	0.059 (3)	0.060 (3)	0.052 (3)	−0.004 (2)	0.005 (2)	−0.001 (2)
C14	0.048 (2)	0.051 (2)	0.054 (3)	−0.005 (2)	0.006 (2)	−0.005 (2)

### Geometric parameters (Å, º)

**Table:**

O1—C8	1.378 (4)	C3—H3	0.9300
O1—C14	1.379 (4)	C4—C5	1.380 (5)
O2—N2	1.212 (4)	C4—H4	0.9300
O3—N2	1.218 (5)	C5—C6	1.376 (5)
N1—C8	1.299 (4)	C5—C8	1.452 (5)
N1—C9	1.396 (5)	C6—C7	1.381 (5)
N2—C12	1.465 (5)	C6—H6	0.9300
C1—C2	1.506 (5)	C7—H7	0.9300
C1—H1A	0.9600	C9—C14	1.387 (5)
C1—H1B	0.9600	C9—C10	1.389 (5)
C1—H1C	0.9600	C10—C11	1.383 (6)
C1—H1D	0.9600	C10—H10	0.9300
C1—H1E	0.9600	C11—C12	1.380 (5)
C1—H1F	0.9600	C11—H11	0.9300
C2—C7	1.373 (6)	C12—C13	1.380 (5)
C2—C3	1.375 (6)	C13—C14	1.366 (5)
C3—C4	1.381 (6)	C13—H13	0.9300
			
C8—O1—C14	104.2 (3)	C5—C4—H4	120.1
C8—N1—C9	104.6 (3)	C3—C4—H4	120.1
O2—N2—O3	122.3 (4)	C6—C5—C4	118.4 (4)
O2—N2—C12	118.5 (4)	C6—C5—C8	121.4 (4)
O3—N2—C12	119.1 (4)	C4—C5—C8	120.2 (4)
C2—C1—H1A	109.5	C5—C6—C7	121.0 (4)
C2—C1—H1B	109.5	C5—C6—H6	119.5
H1A—C1—H1B	109.5	C7—C6—H6	119.5
C2—C1—H1C	109.5	C2—C7—C6	121.0 (4)
H1A—C1—H1C	109.5	C2—C7—H7	119.5
H1B—C1—H1C	109.5	C6—C7—H7	119.5
C2—C1—H1D	109.5	N1—C8—O1	114.8 (3)
H1A—C1—H1D	141.1	N1—C8—C5	128.7 (4)
H1B—C1—H1D	56.3	O1—C8—C5	116.6 (3)
H1C—C1—H1D	56.3	C14—C9—C10	119.5 (4)
C2—C1—H1E	109.5	C14—C9—N1	109.0 (3)
H1A—C1—H1E	56.3	C10—C9—N1	131.4 (4)
H1B—C1—H1E	141.1	C11—C10—C9	117.4 (4)
H1C—C1—H1E	56.3	C11—C10—H10	121.3
H1D—C1—H1E	109.5	C9—C10—H10	121.3
C2—C1—H1F	109.5	C12—C11—C10	120.1 (4)
H1A—C1—H1F	56.3	C12—C11—H11	119.9
H1B—C1—H1F	56.3	C10—C11—H11	119.9
H1C—C1—H1F	141.1	C13—C12—C11	124.4 (4)
H1D—C1—H1F	109.5	C13—C12—N2	117.3 (4)
H1E—C1—H1F	109.5	C11—C12—N2	118.3 (4)
C7—C2—C3	117.7 (4)	C14—C13—C12	113.7 (4)
C7—C2—C1	121.1 (4)	C14—C13—H13	123.1
C3—C2—C1	121.2 (4)	C12—C13—H13	123.1
C2—C3—C4	122.0 (4)	C13—C14—O1	127.8 (4)
C2—C3—H3	119.0	C13—C14—C9	124.8 (4)
C4—C3—H3	119.0	O1—C14—C9	107.4 (4)
C5—C4—C3	119.9 (4)		
			
C7—C2—C3—C4	2.4 (7)	N1—C9—C10—C11	−179.9 (4)
C1—C2—C3—C4	−177.7 (4)	C9—C10—C11—C12	−1.1 (7)
C2—C3—C4—C5	−2.1 (7)	C10—C11—C12—C13	0.8 (7)
C3—C4—C5—C6	0.5 (7)	C10—C11—C12—N2	179.8 (4)
C3—C4—C5—C8	−178.9 (4)	O2—N2—C12—C13	172.0 (4)
C4—C5—C6—C7	0.8 (6)	O3—N2—C12—C13	−5.9 (7)
C8—C5—C6—C7	−179.8 (4)	O2—N2—C12—C11	−7.0 (7)
C3—C2—C7—C6	−1.0 (6)	O3—N2—C12—C11	175.1 (5)
C1—C2—C7—C6	179.0 (4)	C11—C12—C13—C14	0.0 (6)
C5—C6—C7—C2	−0.6 (7)	N2—C12—C13—C14	−179.0 (4)
C9—N1—C8—O1	−0.3 (4)	C12—C13—C14—O1	179.4 (4)
C9—N1—C8—C5	179.6 (4)	C12—C13—C14—C9	−0.6 (6)
C14—O1—C8—N1	0.7 (4)	C8—O1—C14—C13	179.2 (4)
C14—O1—C8—C5	−179.2 (3)	C8—O1—C14—C9	−0.8 (4)
C6—C5—C8—N1	174.5 (4)	C10—C9—C14—C13	0.3 (6)
C4—C5—C8—N1	−6.1 (7)	N1—C9—C14—C13	−179.3 (3)
C6—C5—C8—O1	−5.6 (6)	C10—C9—C14—O1	−179.7 (3)
C4—C5—C8—O1	173.8 (4)	N1—C9—C14—O1	0.7 (4)
C8—N1—C9—C14	−0.2 (4)	C4—C5—C8—N1	−6.1 (7)
C8—N1—C9—C10	−179.8 (4)	C4—C5—C8—O1	173.8 (4)
C14—C9—C10—C11	0.5 (6)	C11—C12—N2—O3	175.1 (5)

### Hydrogen-bond geometry (Å, º)

**Table:**

*D*—H···*A*	*D*—H	H···*A*	*D*···*A*	*D*—H···*A*
C10—H10···O2^i^	0.93	2.61	3.502 (6)	160
C1—H1*B*···O2^ii^	0.96	2.80	3.143 (5)	102

Symmetry codes: (i) −*x*+1/2, −*y*, *z*−1/2; (ii) *x*−1/2, −*y*+1/2, −*z*.
